# Association between Dietary Patterns and the Risk of Hyperemesis Gravidarum

**DOI:** 10.3390/nu15153300

**Published:** 2023-07-25

**Authors:** Wenjie Cheng, Lintian Li, Zhaoqing Long, Xiuxiu Ma, Fangyao Chen, Le Ma, Shunming Zhang, Jing Lin

**Affiliations:** 1 School of Public Health, Xi’an Jiaotong University Health Science Center, Xi’an 710061, China; chengwj20@stu.xjtu.edu.cn (W.C.); lilintian@stu.xjtu.edu.cn (L.L.); lzq3120315022@stu.xjtu.edu.cn (Z.L.); maxiuxiu@stu.xjtu.edu.cn (X.M.); chenfy@xjtu.edu.cn (F.C.); male@mail.xjtu.edu.cn (L.M.); 2 Key Laboratory for Disease Prevention and Control and Health Promotion of Shaanxi Province, Xi’an 710061, China

**Keywords:** hyperemesis gravidarum, dietary patterns, factor analysis, pregnant woman, food-frequency questionnaires

## Abstract

(1) Background: Although studies have suggested that dietary interventions may have potential benefits over conventional medical treatments, research on the association between dietary patterns and hyperemesis gravidarum (HG) in pregnant women is scarce. (2) Methods: To explore the relationship between dietary patterns and the risk of HG, a cross-sectional study was conducted in Xi’an, China from April 2021 to September 2022. Dietary intake was assessed by a semi-quantitative food-frequency questionnaire, and then factor analysis was used to derive dietary patterns. HG was defined as persistent and severe nausea and vomiting with weight loss ≥ 5%, pregnancy-unique quantification of emesis (PUQE) score ≥ 13, or hospitalization due to vomiting. Logistic regression models were used to estimate ORs and 95% CIs for HG according to dietary pattern scores. Stratified analyses and tests for interaction were performed by potential confounders. (3) Results: Of the 3122 pregnant women enrolled, 2515 individuals (mean age: 31.2 ± 3.4 years) were included in the final analysis. In total, 226 (8.9%) pregnant women were identified as having HG. Five dietary patterns were identified. After adjusting for covariates, the highest quartile of the “fish, shrimp and meat” and “egg, milk and water drinking” patterns was associated with a 37% and 58% lower risk of HG compared with the lowest quartile, respectively (*p*-trend < 0.05). Conversely, the highest quartile of the “beverage” pattern was associated with a 64% higher risk of HG compared with the lowest quartile (*p*-trend = 0.02). Furthermore, significant interactions were observed between the “egg, milk and water drinking” pattern and parity, employment status and nutritional supplement use (*p*-interaction < 0.05). (4) Conclusions: A diet rich in eggs, milk, seafood and unprocessed poultry and animal meat may be a protective factor against HG, while a diet high in beverages may be detrimental to HG. These associations may vary by parity, employment status and nutritional supplement use.

## 1. Introduction

Hyperemesis gravidarum (HG) is a severe form of nausea and vomiting of pregnancy (NVP) that persists beyond the first trimester and leads to dehydration, electrolyte imbalances, weight loss, acidosis, hypokalemia, and in some cases, Wernicke encephalopathy (WE). It was estimated that HG affects 0.3% to 10.8% of pregnancies [1]. Studies have shown that HG is associated with an increased risk of maternal morbidity (e.g., anemia, eclampsia, venous thromboembolism, gestational hypertension) [2,3] and adverse birth outcomes (e.g., preterm birth and low birth weight) [4]. Clinical treatment for HG is usually symptomatic and ineffective. Therefore, it is crucial to identify risk factors for hyperemesis gravidarum.

Dietary factors have emerged as a potentially modifiable risk factor for HG that can be easily assessed and intervened by health professionals. However, most studies have only examined the association between single nutrients or foods and HG. For example, previous studies have reported the role of vitamin B1 deficiency in causing poor outcomes for pregnant women with HG [1,5,6], or the use of gingerol and curcumin as natural remedies for HG due to their effects on gastrointestinal motility and antiemetic properties [7]. In addition, other studies have suggested that pregnant women who consumed too much dietary fiber were more likely to suffer from NVP or HG [8]. While elucidative, the human diet is based on overall dietary patterns rather than single nutrients or foods. Dietary patterns capture the combined effects of multiple dietary components [9]. To date, no study has reported the relationship between dietary patterns and HG in pregnant women. Considering that dietary patterns derived by factor analysis could reflect actual eating habits [10], we therefore aimed to identify the major dietary patterns using factor analysis among pregnant women and to investigate their associations with HG risk. The findings of this study may provide a scientific basis for developing targeted dietary recommendations and medical nutrition interventions for HG patients to prevent serious complications and improve birth outcomes.

## 2. Materials and Methods

### 2.1. Study Participants

This cross-sectional study was based on data from the cohort study of “Association between maternal peri-pregnancy drug exposure and birth defects in offspring” (NO.2016YFC1000102-2B) conducted at the Northwest Women’s and Children’s Hospital from April 2021 to September 2022.

Pregnant women aged ≥ 18 years and between 6 to 16 weeks of gestation who agreed to participate in the questionnaire survey were eligible for inclusion. The exclusion criteria were mental or cognitive impairments, inability to cooperate with the survey, other medical causes of nausea and vomiting and refusal to complete the questionnaire.

**Figure 1:**
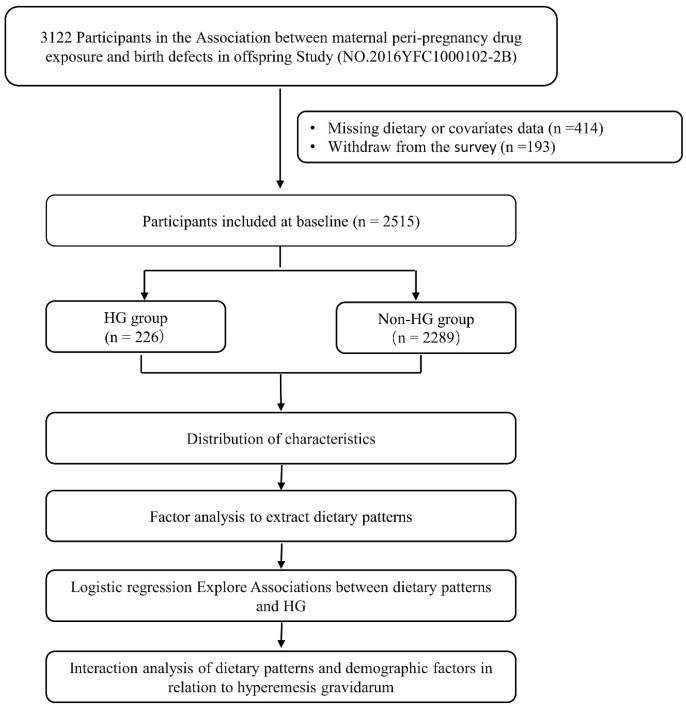
The flow diagram of participant selection and study overview. HG: hyperemesis gravidarum.

A total of 3122 women were enrolled in this study, of whom 414 did not complete the questionnaire and 193 withdrew without completing the questionnaire, resulting in a final sample size of 2515 women (Figure 1). All participants were informed about the research objectives beforehand and signed an informed consent form. The study protocol was approved by the Ethics Committee of the Department of Medicine, Xi’an Jiaotong University (No. 2020-1263).

### 2.2. Pregnancy-Unique Quantification of Emesis (PUQE Questionnaire)

The severity of pregnancy-related nausea and vomiting symptoms was evaluated by PUQE score, which had good internal consistency and reliability (0.846) and validity (0.95) [11,12]. The PUQE score was calculated by summing the scores of three questions related to the frequency of vomiting, the degree of nausea and the degree of retching, with the total score ranging from 0 to 15. Higher scores indicated more severe symptoms and lower quality of life for pregnant women. A total score of ≥13 was indicative of severe NVP.

### 2.3. Diagnostic Criteria for HG [1,13]

1. Ultrasonography verified live birth and early pregnancy between 6–13 weeks of gestation.

2. NVP onset occurs at 6 weeks of gestation and worsens without food intake at 8 weeks of gestation.

3. Loss of >5% of pre-pregnancy body weight and the presence of at least one positive ketonuria test in a random urine specimen.

4. A PUQE score of ≥13 points.

5. Exclude other potential causes of vomiting, such as gastrointestinal or urinary tract infections, viral hepatitis, or pre-existing medical conditions prior to diagnosing HG.

### 2.4. Assessment of Dietary Intake

The dietary intake of the participants was assessed using a semi-quantitative food-frequency questionnaire (FFQ) with a total of 110 food items. The FFQ was adapted from a previously validated FFQ designed for pregnant women in rural western China, with reproducibility and validity coefficients ranging from 0.40 to 0.80 [14,15]. In the adapted version of the FFQ, the participants completed the FFQ by recalling the average frequency and portion size of specific food items consumed over the past year from the date of the survey (around 6–16 weeks pregnant). To assess the effect of long-term (not just during pregnancy) dietary habits on hyperemesis gravidarum, the FFQ used a reference period of the past year instead of the previous 3 months. Daily total energy intake was calculated using data from the FFQ and the 2009 China Food Composition Table (Book 1, 2nd Edition).

### 2.5. Identification of Dietary Pattern Assessment

This study used factor analysis to derive dietary patterns from food groups. Based on nutritional knowledge and food characteristics, food items with similar categories or nutrient composition in the FFQ were combined into food groups. A total of 110 food items in 11 categories in the FFQ were combined into 29 food groups as shown in Table A1. When combining food groups, the sum of the intakes of individual food items in each group indicated the intake of that group, and the units were uniformly converted to g/d for subsequent dietary pattern extraction.

Before constructing dietary patterns, the suitability of the data for factor analysis was checked by calculating the Kaiser–Meyer–Olkin (KMO) statistic and performing Bartlett’s test of sphericity. The KMO value ranged from 0 to 1, and a value ≥0.6 indicated that the data structure was reasonable and suitable for factor analysis [16,17].

**Figure 2:**
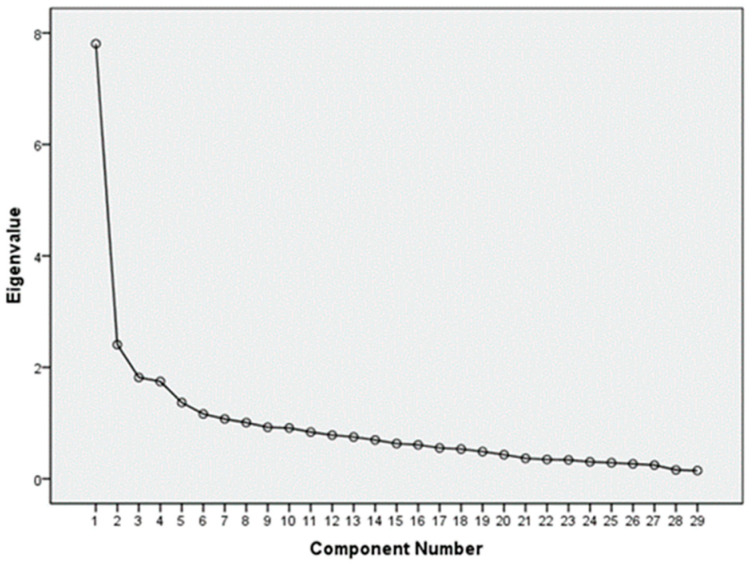
Scree plot of dietary factor analysis of subjects. The abscissa is the component number, and the ordinate is the eigenvalue of dietary common factors. The inflection point occurs at the fifth point, when the cumulative variance rate is 50.4%.

The number of dietary patterns was determined mainly based on eigenvalues, scree plots, cumulative variance explained and factor loadings, while also considering their nutritional interpretability. Eigenvalues > 1 were usually required. The scree plot (Figure 2) shows the eigenvalues on the vertical axis and their ordinal numbers arranged from largest to smallest on the horizontal axis. The extraction of factors was stopped when there was a clear inflection point in the eigenvalues in the plot.

The varimax orthogonal rotation method was used to maximize the sum of variances of each factor loading and to facilitate the interpretation of the extracted dietary patterns. The absolute value of the factor loadings indicates the contribution of each food group to each dietary pattern. The higher the value of a factor loading for a food group in a dietary pattern, the greater its contribution to that pattern. The food groups with factor loadings greater than 0.35 were retained in the composition of each dietary pattern. Each pattern was named according to the 2 to 3 food groups with the highest factor loadings in that pattern, combined with nutritional knowledge.

The score of each dietary pattern as calculated based on the sum of the products of the intake of each food group and its factor loading. The higher the dietary score, the more closely the individual’s dietary intake matched the dietary pattern, while the lower the score, the more different the individual’s dietary intake was from the dietary pattern. The dietary pattern scores were categorized into four groups (Q1–Q4) according to quartiles, from low to high, as the low quartile group (Q1), the middle quartile groups (Q2, Q3) and the high quartile group (Q4).

### 2.6. Assessment of Covariates

The basic information of the participants was collected mainly by questionnaires and electronic medical records. The questionnaires included questions on demographic characteristics (birth year, employment status, education level and annual household income), pregnancy-related information (gestational week, pre-pregnancy weight, parity and nutritional supplements during pregnancy) and personal habits (smoking and drinking).

At the beginning of the study, a trained professional investigator measured the height and weight of the participants at the time of enrollment using a JUMPER weight and height meter (Smart Obstetrics version 2.0). The participants were barefoot and in light clothing. The height and weight were measured with an accuracy of 0.1 cm and 0.1 kg, respectively. The physical activity of the participants was assessed using the International Physical Activity Questionnaire (IPAQ) [18], which consists of seven questions asking about the frequency and duration of vigorous exercise, moderate exercise, walking and sedentary time in the past week. The average daily metabolic equivalent (MET) was also calculated based on the intensity of physical activity.

### 2.7. Statistical Analysis

The general characteristics of the participants in the HG and non-HG groups, and under different dietary patterns, were compared using descriptive statistics. Continuous variables were tested for normality using the Shapiro–Wilk test and Q-Q plots. Normally distributed variables were expressed as mean ± standard deviation and compared using the independent sample *t*-test. Non-normally distributed variables were expressed as median and interquartile range [M (P75-P25)] and compared using the Wilcoxon rank-sum test. Categorical variables were expressed as n (%) and compared using the χ2 test.

The dietary pattern scores were categorized into quartiles (Q1–Q4) from low to high. Logistic regression analyses were used to explore the association between dietary patterns and HG risk, with the lowest scoring group as the reference group to calculate the odds ratio (OR) and 95% confidence interval (CI) for the other three groups. We fitted four models. Model 1 did not control for any variables; model 2 was adjusted for age, gestational week, parity and total energy intake [19,20]; model 3 was further adjusted for physical activity, pre-pregnancy BMI, annual household income, education level, employment status, smoking, alcohol consumption and nutritional supplements; and model 4 was additionally adjusted for other dietary pattern scores. Similar to our previous study [21], we further adjusted for each other dietary pattern score in model 4. The quartiles of dietary pattern scores were also logistically regressed as continuous variables to obtain *p*-values for trends in independent variables and outcome indicators. Moreover, we assessed multicollinearity in model 4 using the variance inflation factor and found no multicollinearity was accepted because all variance inflation factors were less than 2.0.

To examine the heterogeneity of HG risk between groups, we performed subgroup analyses defined by pre-pregnancy BMI, parity, week of gestation, educational level, occupation, annual household income, physical activities and nutritional supplements. In addition, we tested for interactions between these factors and dietary patterns by adding their interaction terms to the final models.

The data were initially entered using Epidata 3.0 software and collected through the Redcap platform. The data were analyzed using SPSS 25.0 statistical software (IBM Corp, Armonk, NY, USA). A two-sided test was used, and *p* < 0.05 was considered statistically significant.

## 3. Results

### 3.1. Characteristics of the Study Participants

Among 2515 pregnant women, 226 were HG and 2289 were non-HG. The age range of the study population was 21 to 45 years with a mean age of 31.2 ± 3.4 years. There were no significant differences between the HG and non-HG groups in terms of demographic characteristics such as age, pre-pregnancy BMI, physical activity, total energy intake, education, employment status, annual household income, parity, nutritional supplements, smoking and alcohol consumption (*p* > 0.05); however, there was a significant difference between the two groups in terms of gestational week (*p* < 0.05), as shown in Table 1.

### 3.2. Types of Dietary Patterns

The suitability test for factor analysis showed that KMO = 0.886 and Bartlett’s sphericity *p* < 0.001, indicating that the data were suitable for factor analysis and that there was a strong correlation among the food groups. In this study, eight factors with eigenvalues > 1 were obtained (Table 2). Based on the inflection point of the scree plot (Figure 2), the factor interpretability and the cumulative variance explained, five factors were extracted as the main dietary patterns in this study, with eigenvalues of 7.806, 2.407, 1.819, 1.746 and 1.367 and contribution rates of 18.899%, 10.338%, 8.486%, 6.528% and 6.152%, respectively, accounting for 50.4% of the total variance. The food groups included in each dietary pattern and their factor loadings are shown in Table 3. The food groups with factor loadings greater than 0.35 were retained in each dietary pattern, and each pattern was named according to the two to three food groups with the highest factor loadings in that pattern and combined with nutritional knowledge.

Factor 1 was named “prudent” dietary pattern because it included a variety of foods beneficial to human health; factor 2 was named the “fish, shrimp and meat” pattern because it featured fish and shrimp meat, non-processed poultry and livestock meat, nuts and yogurt products; factor 3 was named the “sweet and processed meats” dietary pattern because it consisted mainly of processed meats, baked goods and confectionery; factor 4 was named the “beverages” dietary pattern because it consisted mainly of sugar-free colas, sugary drinks and coffee drinks; and factor 5 was named the “egg, milk and water drinking” dietary pattern because it consisted mainly of milk and dairy products, eggs, water and yogurt. The score of each dietary pattern was calculated by summing up the products of the intake of each food group and its factor loading. The higher the dietary score, the more closely the individual’s dietary intake matched the dietary pattern.

### 3.3. Distribution of Characteristics across Different Dietary Patterns

There were significant differences in gestational week (*p* < 0.01), physical activity (*p* = 0.02), total energy intake (*p* < 0.001) and parity (*p* < 0.01) across quartiles of dietary scores in the “prudent” dietary pattern (Table A2).

There were significant differences in total energy intake (*p* < 0.001), employment status (*p* = 0.03), annual household income (*p* < 0.001) and nutritional supplements (*p* = 0.02) across quartiles of dietary scores in the “fish, shrimp and meat” pattern (Table A3).

There were significant differences in age (*p* < 0.001), pre-pregnancy BMI (*p* < 0.01), gestational week (*p* = 0.02), total energy intake (*p* < 0.001) and smoking (*p*< 0.01) across quartiles of dietary scores in the “sweet and processed meats” dietary pattern (Table A4).

There were significant differences in age (*p* < 0.001), total energy intake (*p* < 0.001), education (*p* = 0.03), annual household income (*p* < 0.01), parity (*p* < 0.001), smoking (*p* = 0.02) and alcohol consumption (*p* = 0.03) across quartiles of dietary scores in the “beverages” dietary pattern (Table A5).

There were significant differences in age (*p* < 0.001), total energy intake (*p* < 0.001), physical activity (*p* = 0.02), education (*p* < 0.001), employment status (*p* < 0.01), annual household income (*p* < 0.001) and nutritional supplements (*p* < 0.001) across quartiles of dietary scores in the “egg, milk and water drinking” dietary pattern (Table A6).

### 3.4. Associations between Dietary Patterns and HG

Table 4 shows the associations of five dietary patterns with HG. After the fully adjusted model 4, the risk of HG was lower in pregnant women in the Q4 quartile of the dietary score than in those in the Q1 quartile, and these women were associated with a 7% reduction in risk (OR = 0.93, 95% CI: 0.61, 1.43, *p* = 0.804) in the “prudent” dietary pattern; the risk of HG was lower in pregnant women in the Q4 quartile of the dietary score than in those in the Q1 quartile, with a 37% reduction in the risk of HG (OR = 0.63, 95% CI: 0.41, 0.95, *p* = 0.03) in the “fish, shrimp and meat” pattern; the risk of HG was 9% higher in pregnant women in the Q4 quartile of the dietary score than in those in the Q1 quartile (OR = 1.09, 95% CI: 0.69, 1.71, *p* = 0.65) in the “sweet and processed meats” dietary pattern; the OR for HG risk was 1.64 (95% CI: 1.08, 2.5, *p* = 0.03) for the Q4 quartile compared with the Q1 quartile in the “beverages” dietary pattern; and pregnant women in the Q4 quartile of the dietary score had a 58% lower risk of HG than those in the Q1 quartile (OR = 0.42, 95% CI: 0.26, 0.66, *p* < 0.001) in the “egg, milk and water drinking” dietary pattern. This shows that the *p*-value for trends in all four models was less than 0.05, and the difference was statistically significant in the “fish, shrimp and meat” pattern, “beverages” dietary pattern and “egg, milk and water drinking” dietary pattern.

### 3.5. Subgroup Analysis

Sensitivity analyses were conducted separately for major confounding factors, such as age, pre-pregnancy BMI, parity, gestational week, education level, employment status, annual household income, physical activity and nutritional supplements. There was no significant interaction between different strata of the “prudent” dietary pattern, the “fish, shrimp and meat” pattern, the “sweet and processed meats” dietary pattern and the “beverages” dietary pattern by age, pre-pregnancy BMI, parity, gestational week, education level, employment status, annual household income, physical activity and nutritional supplements (*p* > 0.2 for interaction) (Table A7, Table A8, Table A9 and Table A10). However, there were significant interactions between strata by parity (interaction *p* = 0.03), employment status (interaction *p* = 0.01) and nutritional supplements (interaction *p* = 0.02) in the “egg, milk and water drinking” dietary pattern (Table 5).

In primiparous women, the adjusted OR for HG risk was 0.44 (95% CI: 0.24, 0.83, *p* < 0.01) for the Q4 quartile of the dietary pattern score compared with the Q1 quartile; in multiparous women, the adjusted OR for HG risk was 0.35 (95% CI: 0.18, 0.69, *p* < 0.01) for the Q4 quartile compared with the Q1 quartile. The association was stronger in the multiparous group than in the primiparous group.

Among unemployed women, the adjusted OR for HG risk was 0.19 (95% CI: 0.05, 0.72, *p* < 0.01) for the Q4 quartile of the dietary pattern score compared with the Q1 quartile; among employed women, the adjusted OR for HG risk was 0.46 (95% CI: 0.26, 0.83, *p* < 0.01) for the Q4 quartile compared with the Q1 quartile. The association was stronger in the unemployed group than in the employed group.

Among women who did not take nutritional supplements, the adjusted OR for HG risk was 0.29 (95% CI: 0.14, 0.60, *p* < 0.01) for the Q4 quartile of dietary pattern score compared with the Q1 quartile; among women who took nutritional supplements, the adjusted OR for HG risk was 0.60 (95% CI: 0.27, 1.33, *p* < 0.01) for the Q4 quartile compared with the Q1 quartile. The association was stronger for women who did not take nutritional supplements than for those who took nutritional supplements.

## 4. Discussion

In this cross-sectional study, five main dietary patterns were identified in the pregnant women: “prudent” dietary pattern; “fish, shrimp and meat” dietary pattern; “sweet and processed meat” dietary pattern; “beverage” dietary pattern; and “egg, milk and water drinking” dietary pattern. To the best of our knowledge, our study was the first study that investigated the association between dietary patterns and the risk of HG. Overall, our results indicated that a dietary pattern characterized by a high intake of eggs, milk or dairy products, fish, shrimp, unprocessed poultry, and animal meat, as well as drinking water, was inversely associated with HG, while a diet high in beverages was positively associated with HG. Furthermore, the association was stronger in the multiparous women, unemployed and in those who did not take nutritional supplements. These findings were supported by several previous studies. For example, it was reported that in the first trimester, the meat, seafood and milk consumption in women with NVP was lower both quantitatively and as a proportion of energy compared to non-NVP women [22,23], while women with NVP had a significantly higher intake of sugar-sweetened soft drinks than other pregnant women [24]. Nevertheless, these previous studies focused only on a single food or nutrient; however, the human diet is the sum of food and nutrients. Therefore, this study provides novel evidence for the relationship between dietary patterns and HG.

Our study shows that a ‘fish, shrimp and meat diet’, which is characterized by fish, shrimp, non-processed poultry and livestock meat, as well as nuts and yogurt products, is associated with a reduced risk of HG. Fish and shrimp are rich sources of high-quality animal protein and *n*-3 polyunsaturated fatty acids, and the lack of polyunsaturated fatty acids, such as eicosapentenoic acid (EPA) and docosahexenoic acid (DHA), may be associated with HG [25]. Previous studies have demonstrated that pregnant women without HG consumed more fish and seafood and had slightly higher levels of *n*-3 long-chain fatty acids than pregnant women with HG [23], and that *n*-3 polyunsaturated fatty acids may inhibit the elevation of maternal estrogen levels, which are implicated in the pathogenesis of HG [26]. Secondly, as a common clinical antiemetic, vitamin B6 is rich in pyridoxal phosphate, a coenzyme of numerous aminotransferases and decarboxylases, which promotes the decarboxylation of glutamate and enhances the production of γ-aminobutyric acid, an important inhibitory neurotransmitter in the brain that can exert antiemetic effects [27]. Animal liver, fish, meat and nuts are good sources of vitamin B6 [28,29]. Therefore, increasing the intake of protein-rich foods, such as fish, shrimp, meat and nuts, may have a protective effect against HG.

Our study shows that the “egg, milk and water diet”, which is characterized by milk and dairy products, eggs, water and yoghurt, also has a protective effect on HG. Studies have shown that gastrin is involved in regulating gastric rhythms and correcting disturbances in gastric electromyographic rhythms [30], and that gastrin is mainly secreted by G cells stimulated by protein-based foods [31]. Therefore, the intake of protein-rich foods is important in correcting abnormal peristalsis and reducing pregnancy vomiting. B vitamins are known to stimulate appetite, and vitamin B1 deficiency can cause nausea, vomiting, and anorexia, and the requirement for vitamin B1 increases by 45.5% during pregnancy due to the increased energy demand [1,32]. It has been shown that women with NVP consumed less protein-based foods than those without NVP [22], so egg and milk are beneficial for correcting abnormal gastrointestinal disorders and reducing the incidence of NVP. However, there are also conflicting views that the incidence of NVP is associated with a high intake of eggs and milk [33]. Therefore, this issue needs to be further explored in the future. In addition, our study showed that hydration was also beneficial for preventing NVP, which is consistent with the findings of the Norwegian Mother and Child Cohort Study [23], which showed that drinking one to two glasses of water per day prevented vomiting. As a carrier of nutrients and waste products, adequate water intake facilitates the rapid elimination of potentially emetogenic substances [34]. Clinically, HG patients who received rehydration therapy experienced significant relief from nausea and vomiting [1]. Therefore, adequate water intake is a protective factor against HG.

In contrast to these two dietary patterns that have a protective effect on HG, the “beverage” dietary pattern, which consists mainly of sugar-free cola, sugar-sweetened beverages and coffee drinks, is associated with an increased risk of HG. As a typical carbonated beverage, cola contains a high level of carbon dioxide gas, which can cause gastrointestinal discomfort, such as acid reflux and indigestion [35], and aggravate nausea and vomiting in pregnant women when consumed in large and fast amounts. Coffee contains thiaminase, an enzyme that catalyzes the cleavage of thiamin (vitamin B1), destroying its activity and accelerating the loss from the body [36]. We already know that vitamin B1 is involved in the metabolism of sugars as coenzymes and has a role in maintaining normal digestion, promoting gastrointestinal motility and increasing appetite. Inadequate vitamin B1 before or during pregnancy is undoubtedly an important cause of NVP. Theoretically, pregnant women with NVP or even HG during pregnancy have low energy intake and body weight loss, but some pregnant women with NVP have a paradoxical combination of low body weight and high energy intake [24]. This may be due to their preference for cola, sugary drinks or coffee, which do not provide adequate nutrients for the body to support life and function efficiently [37]. We do not recommend these dietary patterns for pregnant women. This is in line with the 2015–2020 Dietary Guidelines for Americans [38] and the 2022 Dietary Guidelines for Chinese Residents [39], which provide nutritional recommendations for pregnant women.

The “prudent” dietary pattern, which is characterized by fruits and vegetables, potatoes, onions, dark green and white leafy vegetables, whole grains and legumes, mushrooms and algae, soybeans and soy products, fruits, nuts, and refined noodles, is not statistically significant in terms of its protective effect on HG, but it is consistent with the 2015–2020 US Dietary Guidelines [38] and the Chinese Dietary Guidelines 2022 [39]. The “prudent” dietary pattern does not protect against HG, but we still need to follow these guidelines and advocate that pregnant women follow this dietary pattern. We speculate that this may be due to the following reasons: Firstly, whole grains and legumes are rich in dietary fiber [40], and their association with HG risk is not clear. Studies have shown that dietary fiber intake was significantly higher in the NVP group compared to asymptomatic women [24] and that higher dietary fiber intake did not reduce the occurrence of NVP [8], but another study in Finland reported no statistical difference in dietary fiber intake between women with and without NVP [22]. We hypothesize that dietary fiber may delay gastric emptying and may not have a positive effect on improving NVP. Secondly, with the establishment of the placental circulation, the levels of placental ROS and superoxide increase significantly [41], activating cytokines, leading to changes in maternal inflammatory factors and causing oxidative stress [42], and the oxidative imbalance is more severe in HG patients [42,43]. However, as naturally potent antioxidants, flavonoids can inhibit free radical generation, reduce lipid peroxidation, stimulate antioxidant enzymes [44] and have a good contribution to the regulation of oxidative imbalance in HG patients. Coincidentally, garlic, onion and garlic scapes are just good dietary sources of flavonoids [45,46]. It was reported that women who consume large amounts of onion vegetables have a lower risk of severe vomiting during pregnancy [23]. Finally, a dose–response relationship between Helicobacter pylori (Hp) infection and HG has been demonstrated, and garlic and onions, which have antibiotic properties and are rich in allicin, are effective against Gram-negative bacteria, such as Hp [47], and we hypothesized that onion vegetables may alleviate the symptoms of HG by inhibiting Hp infection and have a protective effect against HG.

The “sweet and processed meat” dietary pattern, which is based on processed meat, baked goods and confectionery snacks, was associated with an increased risk of HG, but the difference was not significant. Signorello et al. found that total and saturated fat intake was significantly higher in women with hyperemesis than in women without hyperemesis [48]. Women with higher body fat have higher concentrations of aromatase, an enzyme that converts androgens into estrogens [49], and elevated levels of estrogen are an important factor in the development of HG [50]; foods rich in saturated fat are also rich in cholesterol, a precursor for estrogen synthesis [49]. It has been demonstrated that estradiol levels in HG patients are 26% higher than the mean estradiol levels in controls and that mean levels of sex-hormone-binding globulin are 37% higher [50], and we hypothesized that elevated levels of estrogen may be responsible for the intensity of vomiting during pregnancy and that increased intake of saturated fat may be involved in this pathophysiological process. The incidence of NVP was associated with a high intake of sugar or sweeteners, which is also consistent with a previous study [33].

In the “egg, milk and water drinking” dietary pattern, there were stratified interactions by parity, employment status and nutritional supplements. This study showed that the quartiles of dietary scores were more strongly associated with HG and more sensitive to the “egg, milk and water drinking” dietary pattern in multiparous women, unemployed women and women who did not take nutritional supplements. We speculate that multiparous women may be more sensitive to egg and milk due to their poorer physical condition and protein-storage capacity than primiparous women, and that some micronutrients are also less available than primiparous women, which can aggravate HG symptoms. Egg and milk are rich in micronutrients [51,52], which may be the reason why multiparous women are more sensitive to this dietary pattern. Similarly, women who did not take nutritional supplements before pregnancy have low mineral and vitamin reserves and are more prone to micronutrient deficiencies during pregnancy when the demand is high, so they may be more sensitive to the “egg, milk and water drinking” dietary pattern, which is high in nutrients.

### Study Strengths and Limitations

To our knowledge, this is the first study to investigate the association between dietary patterns and the risk of HG, a wide range of potential confounders were adjusted in our analysis. Overall, the data highlight the beneficial effects of the “egg, milk and water drinking” dietary pattern and the “fish, shrimp and meat” dietary pattern, as well as the harmful effects of the “beverage” dietary pattern on HG risk in pregnant women from northwest China. These findings may help improve HG prevention and make public dietary recommendations for women ready for pregnancy, especially for those women with a family history of HG.

This study also has some limitations. Firstly, the FFQ is not optimal for the measurement of absolute dietary intake, but the use of a dietary pattern approach permitted ranking according to food group intake and so was considered appropriate. Also, the application of the FFQ might result in participants’ misclassification in terms of dietary intake, but the use of an FFQ allowed dietary intake to be captured over a 3-month semester and facilitated the recruitment of a large, geographically diverse sample, albeit a convenient one. Secondly, the dietary patterns, derived using factor analysis, involve some arbitrary decisions, including the consolidation of food items into groups, the number of factors to extract, the rotation method and the naming of the factors. Thirdly, it was not possible to include all potential confounders. Thus, the possibility of residual confounding by factors that have not been evaluated cannot be ruled out. Fourthly, as our participants were mainly from the northwest region of China, there may be differences in food types, dietary structure and lifestyle habits between different regions, and our results may not be generalizable to other populations. Fifthly, some more exhaustive food agreement tests should be carried out in the future. In addition, our study sample included relatively less pregnant women aged ≥ 35 years. Thus, future studies can be carried out with a wider aged population with a large sample. Sixthly, our FFQ used a 1-year reference period rather than 3 months, which mainly aimed to assess the effect of habitual (not just during pregnancy) dietary habits on HG. However, the whole pregnancy duration is 9 months, and previous studies usually assess the dietary intake for the previous 3 months. We acknowledge that there will be changes in food intake due to pregnancy. Thus, future studies are warranted to assess the association between diet during pregnancy and HG. Finally, as an observational study, causal relationships between HG and dietary patterns cannot be inferred, and further randomized controlled trials are needed.

## 5. Conclusions

In conclusion, we found that dietary patterns were associated with HG risk in pregnant women. We recommend that pregnant women increase their consumption of eggs, milk and dairy products, as well as fish, shrimp, and non-processed poultry and livestock meat, as part of a balanced diet and ensure adequate daily water intake. We also suggest that pregnant women reduce or avoid carbonated drinks, sugary drinks and coffee beverages and limit their intake of processed meat and sweets. These findings provide valuable information for the establishment of preventive strategies against HG through dietary modifications in the pregnant population.

## Figures and Tables

**Table 1:** Baseline characteristics of participants in the study.

Characteristic	Non-HG (*n* = 2289)	HG (*n* = 226)	*p*
Age (years)	31.19 ± 3.44	30.96 ± 3.45	0.34
Pre-pregnancy BMI (kg/m^2^)	21.86 ± 3.55	21.71 ± 2.91	0.52
Week of gestation	12 (3.34)	12.17 (1.21)	<0.01
Physical activities (MET/h/d)	14.61 (10.45)	14.61 (11.18)	0.83
Total energy intake (kcal/d)	2565.42 (1527.05)	2676.66 (1547.26)	0.43
Educational level			0.52
Primary school	3 (0.13%)	1 (0.44%)	
Middle school	94 (4.11%)	13 (5.75%)	
High school/technical secondary school	222 (9.7%)	20 (8.85%)	
Junior College	589 (25.73%)	63 (27.88%)	
College	1086 (47.44%)	97 (42.92%)	
Postgraduate and higher	295 (12.89%)	32 (14.16%)	
Occupation			0.51
No	493 (21.56%)	53 (23.45%)	
Yes	1794 (78.44%)	173 (76.55%)	
Annual household income (CNY)			0.26
Under 50,000	250 (10.92%)	27 (11.95%)	
50,000–100,000	557 (24.33%)	62 (27.43%)	
100,000–200,000	838 (36.61%)	82 (36.28%)	
200,000–400,000	490 (21.41%)	43 (19.03%)	
Over 400,000	154 (6.73%)	12 (5.31%)	
Parity			0.65
Primigravida	1302 (56.88%)	125 (55.31%)	
Multipara	987 (43.12%)	101 (44.69%)	
Nutritional supplements use			0.53
No	1406 (62.82%)	131 (60.65%)	
Yes	832 (37.18%)	85 (39.35%)	
Smoke			0.55
No	2216 (96.81%)	217 (96.02%)	
Yes	73 (3.19%)	9 (3.98%)	
Liquor			0.15
No	2207 (96.42%)	222 (98.23%)	
Yes	82 (3.58%)	4 (1.77%)	

**Table 2:** Factor eigenvalues, variance contributions and cumulative variance contributions.

Factors	Eigenvalues	Contribution of Variance (%)	Cumulative Contribution Rate (%)
Factor 1	7.806	18.899	18.899
Factor 2	2.407	10.338	29.237
Factor 3	1.819	8.486	37.724
Factor 4	1.746	6.528	44.252
Factor 5	1.367	6.152	50.403
Factor 6	1.163	4.978	55.381
Factor 7	1.073	4.022	59.403
Factor 8	1.008	4.007	63.410

**Table 3:** Dietary pattern factor loading.

Food Groups	Dietary Pattern				
	Prudent Dietary Pattern	Fish, Shrimp and Meat Dietary Pattern	Sweet and Processed Meat Dietary Pattern	Beverage Dietary Pattern	Egg, Milk and Water Drinking Dietary Pattern
Melon and fruit vegetables	0.879	0.117	0.157	0.008	0.118
Tuber crop	0.832	−0.036	0.079	0.009	0.028
Allium	0.812	0.173	0.062	0.012	0.045
Dark green vegetables and white leafy vegetables	0.800	0.167	0.050	0.019	0.021
Whole grains and mixed legumes	0.789	0.219	0.003	0.035	−0.039
Fungus and algae	0.703	0.312	0.041	0.032	0.017
Soybean and soybean products	0.702	0.265	0.018	0.031	0.215
Fruits	0.496	0.321	0.164	0.009	0.172
Nuts	0.435	0.407	0.109	0.102	0.252
Fish	0.285	0.850	0.011	0.061	−0.038
Shrimp	0.224	0.837	0.083	0.058	0.025
Non-processed poultry meat	0.246	0.665	0.082	0.032	0.126
Non-processed livestock meat	0.330	0.535	0.293	0.022	0.313
Processed meat	0.120	0.045	0.840	−0.034	0.022
Baked food	0.013	0.150	0.835	0.040	0.018
Sweets	0.169	0.063	0.826	0.160	0.024
Sugar-free drinks	−0.002	−0.018	0.048	0.851	−0.052
Sugary drinks	0.176	0.170	0.175	0.747	−0.025
Coffee beverages	−0.042	0.025	−0.038	0.723	0.092
Milk and milk products	0.042	0.072	0.139	−0.007	0.714
Eggs	0.135	0.225	0.008	0.000	0.694
Water	0.035	−0.086	−0.113	0.023	0.525
Yogurt	0.245	0.386	0.318	0.023	0.440
Rice	0.218	0.104	−0.001	0.031	0.107
Crafted noodles	0.406	0.013	0.144	0.016	0.037
Edible vegetable oil	0.045	0.008	−0.028	0.012	0.089
Edible animal oils	−0.008	0.053	0.026	−0.029	−0.089
Liquor	0.018	0.012	0.002	0.036	−0.024
Tea	0.006	0.058	0.096	0.181	0.079

Note: Food groups with factor loading > 0.35 are retained.

**Table 4:** The associations of the five dietary patterns with HG risk.

Dietary Patterns	Quartiles (OR, 95%CI)				Trend *p*-Value
	Q1	Q2	Q3	Q4	
Prudent dietary pattern					
Quartile	(−3.11, −0.39)	(−0.39, −0.16)	(−0.16, 0.15)	(0.15, 16.94)	
N (HG/non-HG)	56/571	53/574	54/575	63/565	
Model 1	1	0.94 (0.64, 1.39)	0.96 (0.65, 1.42)	1.14 (0.78, 1.66)	0.502
Model 2	1	0.91 (0.61, 1.35)	0.91 (0.61, 1.34)	1.05 (0.71, 1.54)	0.818
Model 3	1	0.89 (0.59, 1.35)	0.94 (0.63, 1.40)	0.99 (0.66, 1.48)	0.974
Model 4	1	0.83 (0.55, 1.27)	0.86 (0.56, 1.30)	0.93 (0.61, 1.43)	0.804
Fish, shrimp and meat dietary pattern					
Quartile	(−7.13, −0.32)	(−0.32, −0.13)	(−0.13, 0.10)	(0.10, 18.10)	
N (HG/non-HG)	71/557	57/572	50/578	48/578	
Model 1	1	0.78 (0.54, 1.13)	0.68 (0.46, 0.99)	0.65 (0.44, 0.96)	0.019
Model 2	1	0.79 (0.54, 1.14)	0.70 (0.48, 1.03)	0.65 (0.44, 0.96)	0.023
Model 3	1	0.70 (0.47, 1.03)	0.71 (0.48, 1.05)	0.66 (0.44, 0.99)	0.046
Model 4	1	0.63 (0.42, 0.94)	0.58 (0.38, 0.89)	0.63 (0.41, 0.95)	0.027
Sweet and processed meat dietary pattern					
Quartile	(−3.01, −0.24)	(−0.24, −0.11)	(−0.11, 0.06)	(0.06, 32.64)	
N (HG/non-HG)	45/583	56/572	66/561	59/569	
Model 1	1	1.27 (0.84, 1.91)	1.52 (1.03, 2.27)	1.34 (0.90, 2.01)	0.105
Model 2	1	1.23 (0.82, 1.86)	1.45 (0.98, 2.17)	1.26 (0.83, 1.90)	0.201
Model 3	1	1.32 (0.87, 2.02)	1.45 (0.96, 2.19)	1.25 (0.82, 1.92)	0.270
Model 4	1	1.21 (0.78, 1.86)	1.34 (0.87, 2.05)	1.09 (0.69, 1.71)	0.649
Beverage dietary pattern					
Quartile	(−2.94, −0.27)	(−0.27, −0.19)	(−0.19, −0.02)	(−0.02, 18.31)	
N (HG/non-HG)	45/582	56/573	50/577	75/553	
Model 1	1	1.26 (0.84, 1.90)	1.12 (0.74, 1.70)	1.75 (1.19, 2.58)	0.009
Model 2	1	1.23 (0.81, 1.85)	1.10 (0.72, 1.67)	1.73 (1.17, 2.58)	0.014
Model 3	1	1.15 (0.75, 1.76)	1.06 (0.69, 1.63)	1.74 (1.15, 2.61)	0.015
Model 4	1	1.12 (0.72, 1.73)	1.02 (0.66, 1.59)	1.64 (1.08, 2.50)	0.032
Egg, milk and water drinking dietary pattern					
Quartile	(−4.23, −0.63)	(−0.63, −0.09)	(−0.09, 0.45)	(0.45, 12.85)	
N (HG/non-HG)	77/551	60/568	52/575	37/591	
Model 1	1	0.76 (0.53, 1.08)	0.65 (0.45, 0.94)	0.45 (0.30, 0.67)	<0.001
Model 2	1	0.76 (0.53, 1.09)	0.65 (0.45, 0.95)	0.45 (0.30, 0.68)	<0.001
Model 3	1	0.81 (0.56, 1.18)	0.70 (0.47, 1.03)	0.45 (0.29, 0.70)	<0.001
Model 4	1	0.77 (0.53, 1.13)	0.66 (0.44, 0.98)	0.42 (0.26, 0.66)	<0.001

Note: Q1 and Q4 represent the lowest and highest quartile groups of each dietary pattern factor score, respectively; model 1 was not adjusted for confounders; model 2 was adjusted for age, gestational week, number of births and average total energy intake; model 3 was based on model 2 and adjusted for physical activity, pre-pregnancy BMI, annual household income, education level, employment status, smoking, alcohol consumption and nutritional supplements; model 4 was based on model 3 and further adjusted for other dietary pattern scores under the same scale.

**Table 5:** Stratification analysis of dietary pattern of “egg, milk and water drinking” and HG.

Characteristic	Dietary Pattern Score Quartile				Trend *p*-Value	Interaction *p*-Value
	Q1	Q2	Q3	Q4		
Age (years)						
<35 (*n* = 2108)	1	0.64 (0.42, 0.98)	0.67 (0.44, 1.02)	0.43 (0.27, 0.70)	<0.01	0.54
≥35 (*n* = 407)	1	3.45 (1.01, 11.72)	0.67 (0.18, 2.54)	0.24 (0.04, 1.32)	0.04	
Pre-pregnancy BMI (kg/m^2^)						
<24 (*n* = 2009)	1	0.90 (0.59, 1.37)	0.62 (0.39, 0.98)	0.49 (0.29, 0.80)	<0.01	0.29
≥24 (*n* = 506)	1	0.33 (0.12, 0.94)	0.67 (0.27, 1.70)	0.14 (0.04, 0.50)	<0.01	
Parity						
Primigravida (*n* = 1427)	1	0.94 (0.55, 1.60)	0.77 (0.44, 1.34)	0.44 (0.24, 0.83)	<0.01	0.03
Multipara (*n* = 1088)	1	0.65 (0.37, 1.15)	0.54 (0.30, 1.00)	0.35 (0.18, 0.69)	<0.01	
Week of gestation (weeks)						
<12 (*n* = 1017)	1	0.77 (0.33, 1.83)	0.55 (0.21, 1.40)	0.43 (0.16, 1.20)	0.08	0.42
≥12 (*n* = 1498)	1	0.79 (0.47, 1.33)	0.59 (0.34, 1.02)	0.36 (0.19, 0.66)	<0.01	
Educational level						
Under college (*n* = 1005)	1	0.79 (0.41, 1.54)	0.55 (0.27, 1.15)	0.43 (0.20, 0.95)	0.02	0.71
College and higher (*n* = 1510)	1	0.91 (0.49, 1.66)	0.69 (0.37, 1.30)	0.41 (0.20, 0.83)	<0.01	
Occupation						
No (*n* = 546)	1	0.55 (0.21, 1.44)	0.25 (0.08, 0.84)	0.19 (0.05, 0.72)	<0.01	0.01
Yes (*n* = 1967)	1	0.92 (0.55, 1.52)	0.72 (0.43, 1.20)	0.46 (0.26, 0.83)	<0.01	
Annual household income (CNY 10,000)						
<10 (*n* = 896)	1	0.60 (0.28, 1.28)	0.68 (0.32, 1.45)	0.36 (0.15, 0.85)	0.03	0.5
≥10 (*n* = 1619)	1	0.95 (0.54, 1.66)	0.58 (0.32, 1.07)	0.39 (0.20, 0.76)	<0.01	
Physical activities (MET·h/week)						
<14.6 (*n* = 779)	1	0.78 (0.38, 1.60)	0.57 (0.26, 1.25)	0.32 (0.13, 0.76)	<0.01	0.3
≥14.6 (*n* = 1261)	1	0.84 (0.48, 1.49)	0.61 (0.34, 1.11)	0.48 (0.25, 0.94)	0.02	
Nutritional supplements						
No (*n* = 1537)	1	0.52 (0.29, 0.95)	0.55 (0.30, 1.01)	0.29 (0.14, 0.60)	<0.01	0.02
Yes (*n* = 917)	1	1.29 (0.63, 2.65)	0.62 (0.28, 1.36)	0.60 (0.27, 1.33)	0.06	

Note: BMI (body mass index): weight (kg)/height^2^ (m); OR of the Q1 quartile is for reference; analyses were conducted using the adjusted dietary pattern model 4; smoking and alcohol consumption were very poorly distributed among pregnant women, so no stratified analysis of this factor was conducted.

## Data Availability

Upon reasonable request, data can be obtained from the corresponding author.

## Appendix A

**Table A1:** Food entries included in the 29 food groups included in the factor analysis.

No.	Food Groups	Food Items
1	Crafted noodles	Noodles, steamed buns/steamed twisted roll, steamed stuffed bun/stir-fried crispy cake stuffed with vegetables, dumplings/ravioli, fritters, oil cakes, bread
2	Rice class	Rice, rice flour/rice noodles
3	Whole grains and mixed legumes	Corn, millet, mung beans, red beans, long beans, Chinese long beans, green beans
4	Dark green vegetables and white leafy vegetables	Spinach, leek, rape, cabbage, baby Bok choy, chrysanthemum greens, lettuce, celery, asparagus lettuce, bamboo shoots, wild rice stem, cabbage, purple cabbage, cauliflower, broccoli/mater convolvulus/amaranth, ginger, preserved pickles/salted pickles/fermented water vegetable
5	Melon and fruit vegetables	Lotus roots, tomatoes, fresh peppers, dried peppers, radish/summer radish, carrots, cucumber/eggplant/bitter gourd/luffa, pumpkin, wax gourd, zucchini
6	Allium	Onion, garlic scapes, shallots/spring onion, garlic/garlic sprout
7	Tuber crop	Potatoes, sweet potatoes
8	Soybean and soybean products	Bean sprouts (yellow bean sprout and mung bean sprout), soybeans, black soybeans, tofu, shredded tofu/tofu skin/dried tofu/dried bean curd sticks, soy milk
9	Mushroom and algae	Oyster mushroom/shiitake/needle mushroom, black fungus, kelp, dried nori
10	Fruits	Pear, apples, peaches, plums, oranges, tangerines, grapefruit, mangoes, persimmons, papayas, bananas, watermelons, muskmelon, Hami melon, pineapples, cherry tomatoes, dried dates, grapes, cherries, strawberries, dragon fruit, kiwi fruit
11	Nuts	Peanuts, walnuts and sunflower seeds
12	Livestock meat	Fresh pork, beef, mutton, animal offal (pork intestines)
13	Processed meat	Bacon/sausages/ham sausages
14	Poultry meat	Chicken, duck and goose
15	Fish	Freshwater fish (crucian, carp), saltwater fish (yellow croaker, hairtail)
16	Shrimp	Shrimp
17	Eggs	Eggs, duck eggs
18	Milk and milk products	Milk, powdered milk
19	Yogurt	Yogurt
20	Sugary drinks	Coke, sprite, other sugary beverages, such as seven seasons, iced peaks, orange juice, milk tea
21	Sugar-free drinks	Sugar-free coke
22	Tea drinks	Black tea, green tea
23	Water	Boiled water, pure water
24	Caffeinated drinks	Coffee, ground coffee
25	Liquor	Liquor and spirits, red wine, beer
26	Sweets	Milk candy, fruit candy, chocolate, candies and preserves (hawthorn cake)
27	Baked food	Biscuits, cakes, Chinese snacks (green bean cake)
28	Edible vegetable oil	Peanut oil, soybean oil, corn oil, rapeseed oil, blended oil, sesame oil, salad oil
29	Edible animal oil	Lard

**Table A2:** Demographic characteristics of “prudent” dietary pattern.

Characteristic	Q1	Q2	Q3	Q4	*p*
Age (years)	30.93 ± 3.42	31.31 ± 3.46	31.29 ± 3.53	31.14 ± 3.37	0.17
Pre-pregnancy BMI (kg/m^2^)	21.81 ± 3.33	21.86 ± 3.21	21.77 ± 3.59	21.94 ± 3.81	0.84
Week of gestation	12 (4)	12 (2.84)	12.17 (2.5)	12.17 (2.5)	<0.01
Physical activities (MET/h/w)	14.54 (9.68)	15.72 (11.16)	14.96 (10.72)	13.67 (9.53)	0.02
Total energy intake (kcal/d)	2256.65 (1600.36)	2195.68 (1240.90)	2442.66 (1287.25)	3351.33 (1737.43)	<0.001
Educational level					0.30
Primary school	1 (0.16%)	1 (0.16%)	1 (0.16%)	1 (0.16%)	
Middle school	27 (4.31%)	29 (4.63%)	23 (3.66%)	26 (4.14%)	
High school/technical secondary school	60 (9.57%)	48 (7.66%)	63 (10.02%)	71 (11.31%)	
Junior college	138 (22.01%)	172 (27.43%)	167 (26.55%)	175 (27.87%)	
College	305 (48.64%)	299 (47.69%)	305 (48.49%)	273 (43.47%)	
Postgraduate and higher	96 (15.31%)	78 (12.44%)	70 (11.13%)	82 (13.06%)	
Occupation					0.06
No	115 (18.37%)	138 22.01%)	137 (21.78%)	155 (24.72%)	
Yes	511 (81.63%)	489 (77.99%)	492 (78.22%)	472 (75.28%)	
Annual household income (CNY)					0.28
Under 50,000	70 (11.16%)	61 (9.73%)	72 (11.45%)	72 (11.46%)	
50,000–100,000	143 (22.81%)	157 (25.04%)	161 (25.6%)	158 (25.16%)	
100,000–200,000	214 (34.13%)	223 (35.57%)	243 (38.63%)	239 (38.06%)	
200,000–400,000	150 (23.92%)	146 (23.29%)	117 (18.6%)	119 (18.95%)	
Over 400,000	50 (7.97%)	40 (6.38%)	36 (5.72%)	40 (6.37%)	
Parity					<0.01
Primigravida	387 (61.72%)	368 (58.69%)	327 (51.99%)	343 (54.62%)	
Multipara	240 (38.28%)	259 (41.31%)	302 (48.01%)	285 (45.38%)	
Nutritional supplements					0.54
No	396 (64.71%)	387 (63.24%)	379 (61.33%)	375 (61.27%)	
Yes	216 (35.29%)	225 (36.76%)	239 (38.67%)	237 (38.73%)	
Smoke					0.81
No	607 (96.81%)	603 (96.17%)	611 (97.14%)	608 (96.82%)	
Yes	20 (3.19%)	24 (3.83%)	18 (2.86%)	20 (3.18%)	
Liquor					0.15
No	598 (95.37%)	610(97.29%)	606 (96.34%)	612 (97.45%)	
Yes	29 (4.63%)	17 (2.71%)	23 (3.66%)	16 (2.55%)	

Note: BMI (body mass index): weight (kg)/height^2^ (m).

**Table A3:** Demographic characteristics of “fish, shrimp and meat” dietary pattern.

Characteristic	Q1	Q2	Q3	Q4	*p*
Age (years)	31.25 ± 3.33	31.08 ± 3.24	31.07 ± 3.54	31.27 ± 3.65	0.63
Pre-pregnancy BMI (kg/m^2^)	22.02 ± 3.33	21.81 ± 3.45	21.69 ± 3.39	21.86 ± 3.78	0.42
Week of gestation	12.17 (2.67)	12.17 (2.67)	12 (3.34)	12 (3.54)	0.51
Physical activities (MET/h/w)	15.12 (10.66)	14.9 (9.98)	14.3 (10.24)	14.36 (10.61)	0.14
Total energy intake (kcal/d)	2677.07 (1540.92)	2260.44 (1188.08)	2324.8 (1400.27)	3135.52 (1981.30)	<0.001
Educational level					0.13
Primary school	2 (0.32%)	1 (0.16%)	0 (0%)	1 (0.16%)	
Middle school	29 (4.62%)	28 (4.45%)	21 (3.34%)	27 (4.31%)	
High school/technical secondary school	62 (9.87%)	65 (10.33%)	59 (9.39%)	56 (8.95%)	
Junior college	176 (28.03%)	158 (25.12%)	181 (28.82%)	137 (21.88%)	
College	295 (46.97%)	300 (47.69%)	274 (43.63%)	313 (50%)	
Postgraduate and higher	64 (10.19%)	77 (12.24%)	93 (14.81%)	92 (14.7%)	
Occupation					0.03
No	161 (25.64%)	134 (21.37%)	119 (18.95%)	131 (20.93%)	
Yes	467 (74.36%)	493 (78.63%)	509 (81.05%)	495 (79.07%)	
Annual household income (CNY)					<0.001
Under 50,000	73 (11.62%)	82 (13.04%)	58 (9.24%)	62 (9.9%)	
50,000–100,000	182 (28.98%)	159 (25.28%)	147 (23.41%)	131 (20.93%)	
100,000–200,000	243 (38.69%)	210 (33.39%)	251 (39.97%)	215 (34.35%)	
200,000–400,000	105 (16.72%)	136 (21.62%)	144 (22.93%)	147 (23.48%)	
Over 400,000	25 (3.98%)	42 (6.68%)	28 (4.46%)	71 (11.34%)	
Parity					0.20
Primigravida	335 (53.34%)	359 (57.07%)	360 (57.32%)	371 (59.27%)	
Multipara	293 (46.66%)	270 (42.93%)	268 (42.68%)	255 (40.73%)	
Nutritional supplements					0.02
No	364 (59.09%)	399 (65.3%)	407 (66.18%)	367 (59.97%)	
Yes	252 (40.91%)	212 (34.7%)	208 (33.82%)	245 (40.03%)	
Smoke					0.92
No	608 (96.82%)	609 (96.82%)	609 (96.97%)	603 (96.33%)	
Yes	20 (3.18%)	20 (3.18%)	19 (3.03%)	23 (3.67%)	
Liquor					0.50
No	607 (96.66%)	610 (96.98%)	601 (95.7%)	608 (97.12%)	
Yes	21 (3.34%)	19 (3.02%)	27 (4.3%)	18 (2.88%)	

Note: BMI (body mass index): weight (kg)/height^2^ (m).

**Table A4:** Demographic characteristics of “sweet and processed meat” dietary pattern.

Characteristic	Q1	Q2	Q3	Q4	*p*
Age (years)	31.62 ± 3.4	31.31 ± 3.42	31.02 ± 3.46	30.73 ± 3.45	<0.001
Pre-pregnancy BMI (kg/m^2^)	22.2 ± 3.64	21.6 ± 3.21	21.96 ± 3.59	21.61 ± 3.49	<0.01
Week of gestation	12 (3.67)	12.17 (2.67)	12.17 (2.67)	12.17 (3)	0.02
Physical activities (MET/h/w)	14.52 (10.23)	15.08 (11.13)	14.61 (9.84)	14.54 (10.24)	0.80
Total energy intake (kcal/d)	2476.09 (1453.72)	2210.07 (1382.21)	2519.05 (1382.93)	3142.66 (2098.51)	<0.001
Educational level					0.33
Primary school	3 (0.48%)	0 (0%)	1 (0.16%)	0 (0%)	
Middle school	20 (3.18%)	25 (3.98%)	33 (5.26%)	27 (4.3%)	
High school/technical secondary school	56 (8.92%)	71 (11.31%)	53 (8.45%)	62 (9.87%)	
Junior college	167 (26.59%)	164 (26.11%)	169 (26.95%)	152 (24.2%)	
College	289 (46.02%)	295 (46.97%)	295 (47.05%)	303 (48.25%)	
Postgraduate and higher	93 (14.81%)	73 (11.62%)	76 (12.12%)	84 (13.38%)	
Occupation					0.70
No	135 (21.5%)	141 (22.45%)	142 (22.72%)	127 (20.22%)	
Yes	493 (78.5%)	487 (77.55%)	483 (77.28%)	501 (79.78%)	
Annual household income (CNY)					0.48
Under 50,000	63 (10.03%)	81 (12.9%)	64 (10.21%)	67 (10.67%)	
50,000–100,000	146 (23.25%)	153 (24.36%)	158 (25.2%)	162 (25.8%)	
100,000–200,000	232 (36.94%)	236 (37.58%)	235 (37.48%)	216 (34.39%)	
200,000–400,000	145 (23.09%)	126 (20.06%)	130 (20.73%)	131 (20.86%)	
Over 400,000	42 (6.69%)	32 (5.1%)	40 (6.38%)	52 (8.28%)	
Parity					0.82
Primigravida	354 (56.37%)	358 (57.01%)	348 (55.5%)	365 (58.12%)	
Multipara	274 (43.63%)	270 (42.99%)	279 (44.5%)	263 (41.88%)	
Nutritional supplements					0.20
No	364 (59.28%)	392 (63.74%)	398 (64.93%)	383 (62.58%)	
Yes	250 (40.72%)	223 (36.26%)	215 (35.07%)	229 (37.42%)	
Smoke					<0.01
No	595 (94.75%)	612 (97.45%)	604 (96.33%)	618 (98.41%)	
Yes	33 (5.25%)	16 (2.55%)	23 (3.67%)	10 (1.59%)	
Liquor					0.07
No	600 (95.54%)	615 (97.93%)	609 (97.13%)	602 (95.86%)	
Yes	28 (4.46%)	13 (2.07%)	18 (2.87%)	26 (4.14%)	

Note: BMI (body mass index): weight (kg)/height^2^ (m).

**Table A5:** Demographic characteristics of “beverage” dietary pattern.

Characteristic	Q1	Q2	Q3	Q4	*p*
Age (years)	31.52 ± 3.5	31.38 ± 3.37	31.06 ± 3.45	30.72 ± 3.4	<0.001
Pre-pregnancy BMI (kg/m^2^)	21.93 ± 3.38	21.62 ± 3.44	21.87 ± 3.62	21.95 ± 3.52	0.31
Week of gestation	12 (3.5)	12.17 (2.5)	12.17 (3.34)	12 (3.17)	0.10
Physical activities (MET/h/w)	14.45 (10.64)	14.64 (10.06)	14.91 (10.19)	14.61 (10.92)	0.89
Total energy intake (kcal/d)	2476.09 (1453.72)	2210.07 (1382.21)	2519.05 (1382.93)	3142.66 (2098.51)	<0.001
Educational level					0.03
Primary school	0 (0%)	1 (0.16%)	3 (0.48%)	0 (0%)	
Middle school	31 (4.94%)	26 (4.13%)	20 (3.19%)	28 (4.46%)	
High school/technical secondary school	68 (10.85%)	51 (8.11%)	59 (9.41%)	64 (10.19%)	
Junior college	146 (23.29%)	187 (29.73%)	178 (28.39%)	141 (22.45%)	
College	299 (47.69%)	291 (46.26%)	296 (47.21%)	296 (47.13%)	
Postgraduate and higher	83 (13.24%)	73 (11.61%)	71 (11.32%)	99 (15.76%)	
Occupation					0.96
No	132 (21.05%)	139 (22.13%)	135 (21.57%)	139 (22.13%)	
Yes	495 (78.95%)	489 (77.87%)	491 (78.43%)	489 (77.87%)	
Annual household income (CNY)					<0.01
Under 50,000	78 (12.44%)	72 (11.45%)	70 (11.16%)	55 (8.76%)	
50,000–100,000	143 (22.81%)	180 (28.62%)	161 (25.68%)	135 (21.5%)	
100,000–200,000	228 (36.36%)	219 (34.82%)	239 (38.12%)	233 (37.1%)	
200,000–400,000	125 (19.94%)	121 (19.24%)	127 (20.26%)	159 (25.32%)	
Over 400,000	53 (8.45%)	37 (5.88%)	30 (4.78%)	46 (7.32%)	
Parity					<0.001
Primigravida	323 (51.52%)	352 (55.96%)	353 (56.3%)	397 (63.22%)	
Multipara	304 (48.48%)	277 (44.04%)	274 (43.7%)	231 (36.78%)	
Nutritional supplements					0.27
No	392 (64.37%)	395 (64.12%)	384 (62.54%)	366 (59.51%)	
Yes	217 (35.63%)	221 (35.88%)	230 (37.46%)	249 (40.49%)	
Smoke					0.02
No	613 (97.77%)	614 (97.62%)	606 (96.65%)	596 (94.9%)	
Yes	14 (2.23%)	15 (2.38%)	21 (3.35%)	32 (5.1%)	
Liquor					0.03
No	608 (96.97%)	613 (97.46%)	610 (97.29%)	595 (94.75%)	
Yes	19 (3.03%)	16 (2.54%)	17 (2.71%)	33 (5.25%)	

Note: BMI (body mass index): weight (kg)/height^2^ (m).

**Table A6:** Demographic characteristics of “egg, milk and water drinking” dietary pattern.

Characteristic	Q1	Q2	Q3	Q4	*p*
Age (years)	30.64 ± 3.57	31.28 ± 3.33	31.44 ± 3.47	31.32 ± 3.36	<0.001
Pre-pregnancy BMI (kg/m^2^)	21.9 ± 3.48	21.65 ± 3.83	21.84 ± 3.4	21.98 ± 3.23	0.37
Week of gestation	12 (3.46)	12 (2.67)	12.17 (3.17)	12.17 (3.29)	0.79
Physical activities (MET/h/w)	13.91 (9.27)	14.65 (10.61)	15.09 (11.76)	14.92 (10.19)	0.02
Total energy intake (kcal/d)	2187.15 (1524.95)	2510.66 (1496.98)	2586.98 (1417.22)	2938.8 (1463.70)	<0.001
Educational level					<0.001
Primary school	1 (0.16%)	2 (0.32%)	1 (0.16%)	0 (0%)	
Middle school	42 (6.69%)	32 (5.1%)	14 (2.23%)	17 (2.71%)	
High school/technical secondary school	87 (13.85%)	56 (8.92%)	54 (8.61%)	45 (7.17%)	
Junior college	199 (31.69%)	149 (23.73%)	152 (24.24%)	152 (24.2%)	
College	254 (40.45%)	317 (50.48%)	318 (50.72%)	293 (46.66%)	
Postgraduate and higher	45 (7.17%)	72 (11.46%)	88 (14.04%)	121 (19.27%)	
Occupation					<0.01
No	166 (26.48%)	142 (22.65%)	124 (19.78%)	113 (17.99%)	
Yes	461 (73.52%)	485 (77.35%)	503 (80.22%)	515 (82.01%)	
Annual household income (CNY)					<0.001
Under 50,000	97 (15.4 5%)	58 (9.24%)	67 (10.69%)	53 (8.44%)	
50,000–100,000	170 (27.07%)	167 (26.59%)	152 (24.24%)	130 (20.7%)	
100,000–200,000	226 (35.99%)	227 (36.15%)	224 (35.73%)	242 (38.54%)	
200,000–400,000	99 (15.76%)	132 (21.02%)	145 (23.13%)	156 (24.84%)	
Over 400,000	36 (5.73%)	44 (7.01%)	39 (6.22%)	47 (7.48%)	
Parity					0.49
Primigravida	344 (54.78%)	354 (56.37%)	356 (56.78%)	371 (59.08%)	
Multipara	284 (45.22%)	274 (43.63%)	271 (43.22%)	257 (40.92%)	
Nutritional supplements					<0.001
No	434 (70.92%)	378 (61.46%)	378 (61.56%)	347 (56.61%)	
Yes	178 (29.08%)	237 (38.54%)	236 (38.44%)	266 (43.39%)	
Smoke					0.81
No	608 (96.82%)	608 (96.82%)	609 (97.13%)	604 (96.18%)	
Yes	20 (3.18%)	20 (3.18%)	18 (2.87%)	24 (3.82%)	
Liquor					0.91
No	608 (96.82%)	604 (96.18%)	607 (96.81%)	607 (96.66%)	
Yes	20 (3.18%)	24 (3.82%)	20 (3.19%)	21 (3.34%)	

Note: BMI (body mass index): weight (kg)/height^2^ (m).

**Table A7:** Multi-factorial logistic stratified analysis of “prudent” dietary pattern and HG.

Characteristic	Dietary Pattern Score Quartile				Trend *p*-Value	Interaction *p*-Value
	Q1	Q2	Q3	Q4		
Age (years)						
<35 (*n* = 2108)	1	0.79 (0.50, 1.22)	0.76 (0.48, 1.18)	0.87(0.55, 1.37)	0.54	0.44
≥35 (*n* = 407)	1	3.32 (0.56, 19.54)	3.64 (0.65, 20.51)	2.09 (0.33, 13.21)	0.61	
Pre-pregnancy BMI (kg/m^2^)						
<24 (*n* = 2009)	1	0.89 (0.56, 1.42)	0.88 (0.55, 1.41)	1.01 (0.63, 1.63)	0.97	0.76
≥24 (*n* = 506)	1	0.68 (0.23, 1.96)	0.51 (0.17, 1.54)	0.53 (0.17, 1.70)	0.25	
Parity						
Primigravida (*n* = 1427)	1	0.95 (0.54, 1.68)	0.95 (0.53, 1.70)	1.19 (0.67, 2.10)	0.56	0.42
Multipara (*n* = 1088)	1	0.62 (0.33, 1.18)	0.73 (0.39, 1.34)	0.64 (0.32, 1.27)	0.29	
Week of gestation (weeks)						
<12 (*n* = 1017)	1	3.69 (1.20, 11.31)	3.56 (1.16, 10.94)	4.59 (1.44, 14.63)	0.02	0.90
≥12 (*n* = 1498)	1	0.74 (0.42, 1.31)	0.84 (0.48, 1.48)	0.78 (0.43, 1.40)	0.53	
Educational level						
Under college (*n* = 1005)	1	0.90 (0.42, 1.92)	1.18 (0.55, 2.53)	0.98 (0.44, 2.18)	0.86	0.26
College and higher (*n* = 1510)	1	1.21 (0.63, 2.32)	1.14 (0.60, 2.19)	1.37 (0.69, 2.70)	0.43	
Occupation						
No (*n* = 546)	1	0.95 (0.27, 3.29)	1.21 (0.34, 4.29)	1.57 (0.45, 5.44)	0.37	0.97
Yes (*n* = 1967)	1	1.11 (0.65, 1.91)	1.15 (0.67, 1.97)	1.08 (0.61, 1.91)	0.77	
Annual household income (CNY 10,000)						
<10 (*n* = 896)	1	0.63 (0.25, 1.55)	1.13 (0.50, 2.54)	1.35 (0.58, 3.14)	0.27	0.36
≥10 (*n* = 1619)	1	1.33 (0.72, 2.43)	1.21 (0.65, 2.28)	1.12 (0.58, 2.16)	0.85	
Physical activities (MET·h/w)						
<14.6 (*n* = 779)	1	0.73 (0.31, 1.68)	0.75 (0.34, 1.68)	0.90 (0.40, 2.01)	0.85	0.79
≥14.6 (*n* = 1261)	1	1.24 (0.66, 2.35)	1.34 (0.70, 2.56)	1.39 (0.69, 2.77)	0.36	
Nutritional supplements						
No (*n* = 1537)	1	1.06 (0.54, 2.11)	1.72 (0.89, 3.31)	1.13 (0.56, 2.29)	0.47	0.64
Yes (*n* = 917)	1	1.06 (0.51, 2.21)	0.68 (0.31, 1.51)	1.31 (0.61, 2.79)	0.72	

Note: BMI (body mass index): weight (kg)/height^2^ (m); OR of the Q1 quartile is for reference; analyses were conducted using the adjusted dietary pattern mode l4; smoking and alcohol consumption were very poorly distributed among pregnant women, so no stratified analysis of this factor was conducted.

**Table A8:** Multi-factorial logistic stratified analysis of “fish, shrimp and meat” dietary pattern and HG.

Characteristic	Dietary Pattern Score Quartile				Trend *p*-Value	Interaction *p*-Value
	Q1	Q2	Q3	Q4		
Age (years)						
<35 (*n* = 2108)	1	0.57 (0.36, 0.88)	0.60 (0.38, 0.94)	0.64 (0.41, 1.00)	0.06	0.89
≥35 (*n* = 407)	1	0.65 (0.19, 2.29)	0.27 (0.06, 1.14)	0.36 (0.10, 1.38)	0.07	
Pre-pregnancy BMI (kg/m^2^)						
<24 (*n* = 2009)	1	0.73 (0.46, 1.15)	0.66 (0.41, 1.06)	0.78 (0.49, 1.24)	0.26	0.60
≥24 (*n* = 506)	1	0.24 (0.08, 0.69)	0.27 (0.09, 0.80)	0.21 (0.06, 0.69)	0.01	
Parity						
Primigravida (*n* = 1427)	1	0.64 (0.37, 1.09)	0.49 (0.27, 0.88)	0.57 (0.32, 0.99)	0.03	0.56
Multipara (*n* = 1088)	1	0.57 (0.31, 1.08)	0.66 (0.35, 1.25)	0.67 (0.35, 1.29)	0.29	
Week of gestation (weeks)						
<12 (*n* = 1017)	1	1.06 (0.41, 2.80)	1.86 (0.71, 4.90)	1.62 (0.64, 4.11)	0.20	0.74
≥12 (*n* = 1498)	1	0.58 (0.33, 1.01)	0.46 (0.26, 0.81)	0.55 (0.31, 0.97)	0.02	
Educational level						
Under college (*n* = 1005)	1	0.55 (0.26, 1.19)	0.85 (0.41, 1.78)	0.69 (0.32, 1.49)	0.56	0.63
College and higher (*n* = 1510)	1	0.74 (0.41, 1.35)	0.48 (0.24, 0.94)	0.72 (0.39, 1.35)	0.18	
Occupation						
No (*n* = 546)	1	0.26 (0.07, 0.94)	0.73 (0.25, 2.16)	0.70 (0.23, 2.09)	0.78	0.35
Yes (*n* = 1967)	1	0.80 (0.48, 1.33)	0.61 (0.35, 1.06)	0.72 (0.42, 1.25)	0.15	
Annual household income (CNY 10,000)						
<10 (*n* = 896)	1	0.58 (0.27, 1.26)	1.16 (0.53, 2.51)	0.57 (0.24, 1.33)	0.46	0.78
≥10 (*n* = 1619)	1	0.70 (0.38, 1.28)	0.44 (0.23, 0.85)	0.77 (0.42, 1.40)	0.24	
Physical activities (MET·h/w)						
<14.6 (*n* = 779)	1	0.52 (0.24, 1.13)	0.44 (0.19, 1.01)	0.48 (0.21, 1.06)	0.07	0.18
≥14.6 (*n* = 1261)	1	0.73 (0.40, 1.32)	0.80 (0.43, 1.50)	0.94 (0.51, 1.73)	0.86	
Nutritional supplements						
No (*n* = 1537)	1	0.55 (0.29, 1.03)	0.51 (0.26, 1.00)	0.80 (0.41, 1.53)	0.43	0.60
Yes (*n* = 917)	1	0.86 (0.41, 1.78)	0.84 (0.39, 1.78)	0.67 (0.32, 1.41)	0.31	

Note: BMI (body mass index): weight (kg)/height^2^ (m); OR of the Q1 quartile is for reference; analyses were conducted using the adjusted dietary pattern model 4; smoking and alcohol consumption were very poorly distributed among pregnant women, so no stratified analysis of this factor was conducted.

**Table A9:** Multi-factorial logistic stratified analysis of “sweet and processed meat” dietary pattern and HG.

Characteristic	Dietary Pattern Score Quartile				Trend *p*-Value	Interaction *p*-Value
	Q1	Q2	Q3	Q4		
Age (years)						
<35 (*n* = 2108)	1	1.32 (0.82, 2.11)	1.36 (0.85, 2.18)	1.15 (0.70, 1.88)	0.63	0.52
≥35 (*n* = 407)	1	0.64 (0.17, 2.39)	0.78 (0.22, 2.79)	0.43 (0.10, 1.89)	0.34	
Pre-pregnancy BMI (kg/m^2^)						
<24 (*n* = 2009)	1	1.14 (0.71, 1.84)	1.20 (0.74, 1.93)	1.09 (0.67, 1.79)	0.71	0.64
≥24 (*n* = 506)	1	1.72 (0.49, 6.02)	2.42 (0.73, 8.06)	1.07 (0.27, 4.25)	0.78	
Parity						
Primigravida (*n* = 1427)	1	1.19 (0.64, 2.20)	1.94 (1.08, 3.47)	1.13 (0.60, 2.14)	0.37	0.80
Multipara (*n* = 1088)	1	1.23 (0.65, 2.31)	0.80 (0.41, 1.57)	1.03 (0.54, 2.00)	0.29	
Week of gestation (weeks)						
<12 (*n* = 1017)	1	0.89 (0.33, 2.38)	1.03 (0.40, 2.68)	0.81 (0.31, 2.13)	0.74	0.55
≥12 (*n* = 1498)	1	1.37 (0.76, 2.50)	1.73 (0.97, 3.10)	1.17 (0.61, 2.22)	0.47	
Educational level						
Under college (*n* = 1005)	1	1.37 (0.61, 3.07)	1.79 (0.81, 3.94)	1.23 (0.52, 2.91)	0.54	0.94
College and higher (*n* = 1510)	1	1.13 (0.59, 2.15)	1.45 (0.77, 2.74)	1.03 (0.52, 2.03)	0.73	
Occupation						
No (*n* = 546)	1	0.53 (0.17, 1.68)	0.61 (0.20, 1.91)	0.81 (0.26, 2.49)	0.84	0.36
Yes (*n* = 1967)	1	1.55 (0.88, 2.75)	1.87 (1.08, 3.24)	1.16 (0.63, 2.13)	0.51	
Annual household income (CNY 10,000)						
<10 (*n* = 896)	1	0.76 (0.33, 1.76)	1.09 (0.49, 2.41)	0.78 (0.33, 1.86)	0.83	0.97
≥10 (*n* = 1619)	1	1.36 (0.72, 2.60)	1.69 (0.90, 3.17)	1.22 (0.62, 2.41)	0.47	
Physical activities (MET·h/w)						
<14.6 (*n* = 779)	1	0.84 (0.37, 1.88)	0.90 (0.40, 2.03)	0.76 (0.33, 1.78)	0.59	>0.99
≥14.6 (*n* = 1261)	1	1.51 (0.78, 2.93)	2.00 (1.06, 3.77)	1.42 (0.70, 2.86)	0.22	
Nutritional supplements						
No (*n* = 1537)	1	1.51 (0.76, 2.98)	1.61 (0.83, 3.14)	0.79 (0.37, 1.70)	0.60	0.30
Yes (*n* = 917)	1	1.07 (0.50, 2.30)	1.45 (0.69, 3.05)	1.44 (0.68, 3.07)	0.25	

Note: BMI (body mass index): weight (kg)/height^2^ (m); OR of the Q1 quartile is for reference; analyses were conducted using the adjusted dietary pattern model 4; smoking and alcohol consumption were very poorly distributed among pregnant women, so no stratified analysis of this factor was conducted.

**Table A10:** Multi-factorial logistic stratified analysis of “beverage” dietary pattern and HG.

Characteristic	Dietary Pattern Score Quartile				Trend *p*-Value	Interaction *p*-Value
	Q1	Q2	Q3	Q4		
Age (years)						
<35 (*n* = 2108)	1	1.10 (0.69, 1.77)	1.00 (0.62, 1.61)	1.60 (1.02, 2.53)	0.06	0.48
≥35 (*n* = 407)	1	1.04 (0.29, 3.76)	0.89 (0.22, 3.70)	2.26 (0.64, 7.93)	0.25	
Pre-pregnancy BMI (kg/m^2^)						
<24 (*n* = 2009)	1	1.11 (0.69, 1.79)	0.99 (0.61, 1.62)	1.72 (1.08, 2.75)	0.04	0.26
≥24 (*n* = 506)	1	1.46 (0.46, 4.62)	1.27 (0.41, 3.93)	1.34 (0.42, 4.26)	0.70	
Parity						
Primigravida (*n* = 1427)	1	1.15 (0.63, 2.12)	0.92 (0.49, 1.72)	1.53 (0.86, 2.73)	0.21	0.22
Multipara (*n* = 1088)	1	1.11 (0.59, 2.09)	1.11 (0.59, 2.11)	1.74 (0.92, 3.29)	0.12	
Week of gestation (weeks)						
<12 (*n* = 1017)	1	2.49 (0.78, 7.94)	2.43 (0.76, 7.77)	8.49 (2.84, 25.36)	<0.001	0.60
≥12 (*n* = 1498)	1	0.74 (0.42, 1.30)	0.69 (0.39, 1.21)	0.86 (0.49, 1.53)	0.57	
Educational level						
Under college (*n* = 1005)	1	1.36 (0.61, 3.05)	1.21 (0.53, 2.75)	2.27 (1.02, 5.08)	0.06	0.59
College and higher (*n* = 1510)	1	0.74 (0.38, 1.41)	0.75 (0.39, 1.42)	1.19 (0.64, 2.21)	0.60	
Occupation						
No (*n* = 546)	1	0.42 (0.13, 1.36)	0.52 (0.16, 1.63)	1.38 (0.49, 3.85)	0.51	0.88
Yes (*n* = 1967)	1	1.11 (0.64, 1.94)	1.03 (0.58, 1.81)	1.46 (0.84, 2.55)	0.23	
Annual household income (CNY 10,000)						
<10 (*n* = 896)	1	0.87 (0.38, 2.00)	1.10 (0.49, 2.50)	1.42 (0.61, 3.29)	0.34	0.1
≥10 (*n* = 1619)	1	0.86 (0.46, 1.63)	0.80 (0.42, 1.54)	1.35 (0.74, 2.46)	0.32	
Physical activities (MET·h/w)						
<14.6 (*n* = 779)	1	1.00 (0.41, 2.42)	1.50 (0.66, 3.38)	2.19 (0.97, 4.92)	0.03	0.24
≥14.6 (*n* = 1261)	1	0.89 (0.49, 1.63)	0.65 (0.34, 1.24)	1.30 (0.70, 2.39)	0.62	
Nutritional supplements						
No (*n* = 1537)	1	0.61 (0.29, 1.27)	1.33 (0.70, 2.53)	1.90 (1.00, 3.60)	0.01	0.89
Yes (*n* = 917)	1	1.41 (0.69, 2.87)	0.52 (0.23, 1.20)	1.17 (0.55, 2.48)	0.64	

Note: BMI (body mass index): weight (kg)/height^2^ (m); OR of the Q1 quartile is for reference; analyses were conducted using the adjusted dietary pattern model 4; smoking and alcohol consumption were very poorly distributed among pregnant women, so no stratified analysis of this factor was conducted.
